# Recombinant scorpion toxins: Focus on four-disulfide peptide blockers of Kv1-channels

**DOI:** 10.1080/21655979.2017.1373530

**Published:** 2017-09-21

**Authors:** Oksana Nekrasova, Sergey Yakimov, Mikhail Kirpichnikov, Alexey Feofanov

**Affiliations:** a Bioengineering Department, Biological Faculty, Lomonosov Moscow State University, Moscow, Russia; b Shemyakin-Ovchinnikov Institute of Bioorganic Chemistry, Russian Academy of Sciences, Moscow, Russia

**Keywords:** disulfide rich peptide, potassium channel blocker, recombinant peptide, scorpion toxins, TEV protease activity

## Abstract

We have recently developed a simple and effective bioengineering approach to large-scale production of alpha-KTx, peptide toxins from scorpion venoms, that block voltage-gated potassium channels with high affinity and specificity. This approach was successfully approved for different peptides containing three disulfide bonds. To extend this method to production of peptide toxins with four disulfide bridges, in particular, maurotoxin and hetlaxin, appropriate conditions of a cleavage reaction with tobacco etch virus (TEV) protease need to be found. For this, the interplay between efficiency of TEV hydrolysis and sensitivity of the target peptides to disulfide reducing agents was studied, and optimized protocols of TEV cleavage reaction were worked out. Maurotoxin and hetlaxin were produced in a folded form avoiding *in vitro* renaturation step with yields of 14 and 12 mg/liter of culture, respectively.

Peptide toxins from scorpion venoms, which are specific and high-affinity pore blockers of voltage-gated (Kv) potassium channels are widely used as valuable molecular tools to study pharmacology of Kv channels and the structure of their pore domains. Moreover, peptide blockers have potential medical applications since Kv channels are involved in pathogenesis of some neurological, cardiac, autoimmune and oncological diseases.^1^

Recently we have developed an effective and robust bioengineering approach to high-level production (the yields of 12–22 mg per liter of *E.coli* culture) of fully folded and functionally active recombinant peptides with three disulfide bonds from α-KTx family of scorpion toxins.^2^ The approach is based on bacterial expression of the target peptide fused to maltose binding protein (MBP) and subsequent hydrolysis of the fusion protein with tobacco etch virus (TEV) protease. Under optimized expression conditions MBP fusion proteins were over-expressed in the soluble form (>90%) in *E.coli* cytoplasm with yields about 180–350 mg/l of culture. The target peptides acquired proper fold during cultivation, thus, eliminating the need for renaturation procedure *in vitro*. Non-canonical TEV protease cleavage sites were used in expression cassettes to obtain recombinant peptides with natural primary structure. Decreased efficiency of TEV protease cleavage of some fusion proteins including those with non-canonical TEV sites was overcome using excess of TEV protease without byproduct formation.

The main bottleneck of the developed approach that can impose limitations on the production of disulfide-rich polypeptides is the step of TEV protease hydrolysis of MBP fusion proteins, in which dithiothreitol (DTT), a strong disulfide reducing agent is used to maintain enzymatic activity of the protease. However, increased sensitivity of some three-disulfide toxins (for example, margatoxin and agitoxin 2) to DTT can be successfully overcome by reducing DTT concentration in TEV protease cleavage reaction from 1 to 0.5 mM without noticeable change in enzymatic activity.

Aiming to extend the developed approach^2^ to production of recombinant peptides with 4 disulfide bonds such as hetlaxin (HTX)^3^ and maurotoxin (MTX)^4^ (both from α-KTx 6 subfamily), we constructed plasmids pET-23d-MalE-HTX and pET-23d-MalE-MTX for expression of the target peptides. It was done using the expression vector pET-23d-MalE, which was described previously.^2^ DNA fragments encoding HTX or MTX flanked by N-terminal TEV cleavage site (CS_TEV_) were obtained by a polymerase chain reaction using synthetic oligonucleotides, and then each fragment was cloned into the expression vector as described earlier.^2^

Nucleotide sequences encoding CS_TEV_-toxin fragments were the following (CS_TEV_ coding sequences are shown in bold; stop-codons are shown in italic):

5′- **GAA AAC CTG TAT TTT CAG ATC** AGC TGC ACC GGT TCT AAA CAG TGT TAT GAT CCG TGT AAG AAG AAG ACC GGT TGC CCA AAC GCG AAA TGC ATG AAC AAA AGC TGT AAA TGC TAC GGT TGT *TAA* -3′, for CS_TEV_-HTX;

5′- **GAA AAC CTG TAT TTT CAG GTG** TCT TGC ACC GGC AGC AAA GAT TGC TAT GCG CCG TGC CGG AAA CAG ACC GGC TGC CCG AAC GCG AAA TGC ATT AAC AAA AGC TGC AAA TGC TAT GGC TGC *TAA* -3′, for CS_TEV_-MTX.

Recombinant HTX and MTX were produced in *E.coli* in the form of MBP fusion proteins, MBP-L1-CS_TEV_-HTX and MBP-L1-CS_TEV_-MTX, respectively. MBP and CS_TEV_-toxin moieties were separated by the 42-amino acid L1 linker^2^ that contained His-tag. N-terminal residue of HTX (Ile) or MTX (Val) was located in the next position after the peptide bond cleaved by the TEV protease.

Applying previously developed protocols,^2^ we found that during TEV protease hydrolysis of the fusion proteins, a set of partially or fully denatured HTX- (MTX-) related species were generated even at 0.5 mM DTT (Fig. 1A, E). Further reduction in DTT concentration to 0.1 mM restored production of renatured MTX and HTX, however, efficiency of hydrolysis of both fusion proteins decreased to 30–40% (data not shown).

**Figure 1.**
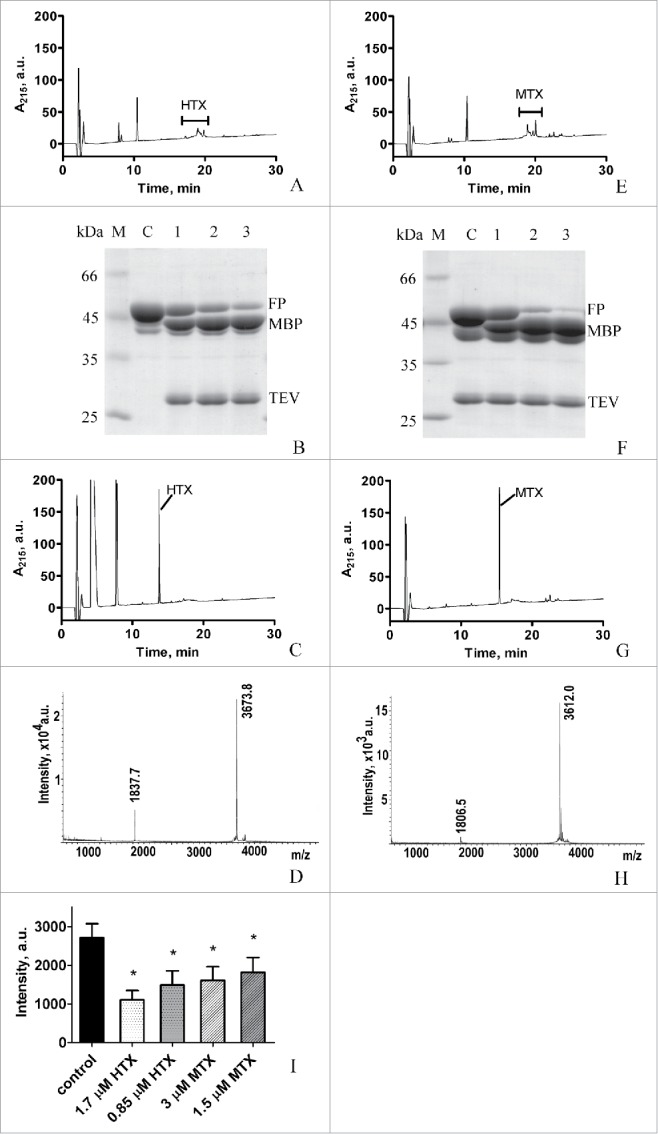
Optimization of the production of recombinant HTX and MTX. A. Hydrolysis of MBP-L1-CS_TEV_-HTX in the presence of 0.5 mM DTT. Analysis with reverse-phase HPLC. All the hydrolysis reactions (A-C, E-G) were performed in the 100 μl volume with 1 mg/ml of MBP-L1-CS_TEV_-HTX (or MBP-L1-CS_TEV_-MTX) and 0.4 mg/ml of TEV protease at 20°C for 16 h. B. Hydrolysis of MBP-L1-CS_TEV_-HTX in the presence of 3 mM GSH/0.3 mM GSSG (lane 1), 6 mM GSH/0.3 mM GSSG (lane 2), and 8 mM GSH/0.4 mM GSSG (lane 3). Analysis with 12% SDS-PAGE. C – control without TEV protease. M – protein molecular weight markers. FP is MBP-L1-CS_TEV_-HTX. C. Hydrolysis of MBP-L1-CS_TEV_-HTX in the presence of 8 mM GSH/0.4 mM GSSG. Analysis with reverse-phase HPLC. Instead of several forms of denatured HTX with retention time near 20 min (A) a peak of renatured HTX peptide appeared at 13.8 min (C). D. MALDI-MS spectrum of renatured HTX. E. Hydrolysis of MBP-L1-CS_TEV_-MTX in the presence of 0.5 mM DTT. Analysis with reverse-phase HPLC. F. Hydrolysis of MBP-L1-CS_TEV_-MTX in the presence of 0.1 (lane 1), 0.2 (lane 2), and 0.3 mM (lane 3) TCEP. Analysis with 12% SDS-PAGE. C – control without TCEP. M – protein molecular weight markers. FP is MBP-L1-CS_TEV_-MTX. G. Hydrolysis of MBP-L1-CS_TEV_-MTX in the presence of 0.2 mM TCEP. Analysis with reverse-phase HPLC. Instead of several forms of denatured MTX with retention time near 20 min (D) a peak of renatured MTX appeared at 15.4 min (F). H. MALDI-MS spectrum of renatured MTX. I. Analysis of activity of renatured HTX and MTX. Renatured HTX and MTX displace competitively fluorescent agitoxin 2 (4 nM) from the Kv1.3 binding site of KcsA-Kv1.3 channel embedded in spheroplast membrane. Control – equivalent amount of a buffer was added to spheroplasts instead of HTX or MTX. Average fluorescence signal intensity of fluorescent agitoxin 2 per cell is presented as a mean of three independent experiments (mean ± SD). Asterisks mark values that are significantly (p < 0.05) different from the control value according to unpaired two-tailed t-test.

With a view to preserve high yields of production for 4-disulfide peptides, we studied the efficiency of TEV protease hydrolysis of MBP fusion proteins and formation of fully folded peptides using alternative reducing agents, namely, Tris-(2-carboxyethyl)-phosphine (TCEP) or a pair of oxidized (GSH) and reduced (GSSG) glutathiones as recommended in.^5^ Both fusion proteins, MBP-L1-CS_TEV_-MTX and MBP-L1-CS_TEV_-HTX, were poorly hydrolyzed in the presence of 3 mM GSH/0.3 mM GSSG. However, the raise of GSH concentration up to 6 or 8 mM (at GSH/GSSG ratio of 20/1) resulted in almost fully hydrolyzed MBP-L1-CS_TEV_-HTX as revealed with denaturing polyacrylamide gel electrophoresis in 12% sodium dodecyl sulfate (SDS-PAGE, Fig. 1B). Moreover, renatured form of HTX is retained under these reaction conditions as follows from appearance of a narrow peak with the 13.8 min retention time in a reverse phase HPLC chromatogram (Fig. 1C). According to data of matrix-assisted laser desorption ionization mass-spectrometry (MALDI-MS) the substance from this peak has a molecular weight (m.w.) of 3673.8 Da that corresponds exactly to m.w. of HTX with 4 disulfide bonds (Fig. 1D).

In the case of MBP-L1-CS_TEV_-MTX, increased GSH concentrations improved the rate of hydrolysis only to a small extent, while complete hydrolysis of the fusion protein was achieved using low concentrations (0.2 – 0.5 mM) of strong reducing agent TCEP (Fig. 1F). At the same time MTX was properly folded at only 0.2–0.3 mM TCEP concentrations and denatured completely at 0.5 mM TCEP. Renatured MTX was characterized by a peak with the 15.4 min retention time in the reverse phase HPLC chromatogram (Fig. 1G) and m.w. of 3612 Da (Fig. 1H). This molecular weight is a characteristic of MTX with four S-S bonds. Finally, 0.2 mM TCEP was successfully used by us to produce almost quantitatively recombinant MTX in a renatured form after TEV protease hydrolysis. HTX turned to be more sensitive to TCEP, and 30 – 40% of the peptide denatured even at 0.2 mM TCEP.

HTX is a new Kv1.3 channel blocker from the scorpion *Heterometrus laoticus*.^3^ MTX is a high affinity blocker of Kv1.2 and K_Ca_ channels^4^ as well as a moderate affinity blocker of Kv1.3 channel.^6,7^ To confirm correct folding of recombinant HTX and MTX, we have tested their ability to bind to the Kv1.3-channel binding site formed in the hybrid KcsA-Kv1.3 channel. To do this, a bioengineering system was used, which was based on a KcsA-Kv1.3 channel embedded in the membrane of spheroplasts and fluorescently labeled peptide blocker agitoxin 2.^8,9^ This system is a reliable analytical tool to search for Kv1.3 blockers in complex mixtures and among individual compounds as well as to characterize their activities.^3,9^ Using this system as described elsewhere,^2,3,8^ we have observed displacement of fluorescent agitoxin 2 from the Kv1.3-channel binding site by HTX and MTX (Fig. 1I). Such competition between HTX (MTX) and agitoxin 2 for the binding to the KcsA-Kv1.3 channel can occur only if the tested peptide has a right secondary structure with correctly formed S-S bonds. Rather high concentration of HTX or MTX was required to displace agitoxin 2 from the KcsA-Kv1.3 channel because affinity of agitoxin 2 to the Kv1.3 binding site was much higher^9^ than that of the tested peptides in accordance with the properties of wild type HTX^3^ and MTX.^4^

In conclusion, we have demonstrated that a previously developed approach^2^ can be successfully adapted for the high-yield production of 4-disulfide α-KTx such as MTX and HTX. MTX and HTX were produced in a recombinant form for the first time, and the yields of folded recombinant peptides were 14 and 12 mg/liter of culture, respectively.

## Abbreviations

DTT: dithiothreitol
GSH: reduced glutathione
GSSG: oxidized glutathione
HTX: hetlaxin
MBP: maltose binding protein
MTX: maurotoxin
TEV: tobacco etch virus
TCEP: Tris-(2-carboxyethyl)-phosphine
